# Consumption of Food Offered by Delivery Applications (Apps)

**DOI:** 10.3390/ijerph21050568

**Published:** 2024-04-29

**Authors:** Jamile Carvalho Tahim, Sara Maria Moreira Lima Verde, Carla Soraya Costa Maia, Ilana Nogueira Bezerra

**Affiliations:** 1 Postgraduate Program in Public Health, Universidade Estadual do Ceará, Fortaleza 60714-903, Brazil; jamile.tahim@aluno.uece.br; 2 Postgraduate Program in Nutrition and Health, Universidade Estadual do Ceará, Fortaleza 60714-903, Brazil; sara.maria@uece.br (S.M.M.L.V.); carla.maia@uece.br (C.S.C.M.)

**Keywords:** food consumption, digital food environment, food services

## Abstract

This study aimed to investigate whether the use of food delivery applications is related to food consumption practices. Methods: Data collection was carried out between 5 and 20 June 2020 in a Brazilian capital with a final sample of 2113 individuals. The instrument included questions about socioeconomic characteristics, anthropometric data, and information about eating practices and the use of delivery apps. Logistic regression models were developed with the consumption of each food group as the outcome variable and the use of the application as the exposure variable. Results: Of those interviewed, 78.1% used delivery apps to purchase food. The frequencies of the consumption of foods considered markers of healthy eating were higher for individuals who did not use the applications (59.7% vs. 38.7% for the daily consumption of fresh fruit, respectively, *p* < 0.0001). The frequencies of the consumption of items considered markers of unhealthy eating were higher for individuals who used apps to purchase food prepared outside the home compared to those who did not (53.7 vs. 38.1 for weekly consumption of hamburgers, respectively, *p* < 0.0001). Conclusion: The use of delivery applications influenced the users’ eating practices through the acquisition of food within the digital scope and is related to a greater frequency of the consumption of unhealthy items by users.

## 1. Introduction

The concept of the digital food environment (DFE) encompasses digital components that can be part of food environments and influence health and nutrition outcomes [1]. Digital food environments comprise digital actors, such as governments, universities, the food industry, and influencers, who carry out activities within this scope (such as digital health promotion, digital food marketing, and information sharing) on digital platforms (such as websites, blogs, and smartphone applications) [2].

In recent years, electronic tools, systems, and devices such as social media, food delivery apps, and food chain websites have gained more space in individuals’ routines as part of the set of influences that represent the digital food environment [1,2].

It is known that food culture is influenced by factors that go beyond individual factors in food choices, thus culminating in an environment in which one is inserted with social and cultural practices (social environment, physical environments, media, guidelines, and food systems) that shape eating behavior. Thus, changes in food environments have an impact on individuals’ behavior and eating patterns and, therefore, affect health and well-being [3].

Moreover, considering that the process of food choices frequently occurs in individuals’ daily lives, it becomes important to study changes in eating practices and the different determinants of choices related to eating behavior, taking into account the context of the increasing use of delivery apps for purchasing food [1,2].

Food retail activities through delivery applications started to integrate the DFE with the expansion of internet access and the dissemination of digital culture with the increased use of mobile devices; moreover, the dynamics of food choices in the digital scope differs from the experience of food acquisition in physical environments [4,5].

In this sense, the aim of this study is to investigate whether the use of food delivery applications for ordering food prepared outside the home is related to food consumption practices.

The originality of this study is that it provides information about changes in eating patterns when the digital food app environment was increasing exponentially, which was during the period of social isolation and when individuals began to show greater engagement with app advertising and product sales strategies.

## 2. Materials and Methods

A cross-sectional study was developed through the application of a population survey with residents of the city of Fortaleza, a metropolis located in the northeast of Brazil, the capital of the state of Ceará, from 5 to 20 June 2020.

The sample population consisted of individuals over 18 years of age. The sample size estimate, based on the sample calculation for a finite population, was 1920 individuals, considering the population size criteria estimated at 2,669,342 individuals according to data from the Brazilian Institute of Geography and Statistics (IBGE) for the year 2020, 50% prevalence of purchasing food outside the home (strategy to maximize the sample size), and adopting a 95% confidence interval, a maximum error of 2.5%, and non-response rate of 20%.

A total of 2430 individuals answered the questionnaire. Of these, 9 did not complete the survey, 8 individuals did not live in Fortaleza, 6 were under 18 years of age, and 294 were duplicate responses. Thus, the total number of eligible and included individuals was 2113 individuals.

The individuals were invited to participate in the research using the non-probability sampling technique called ‘snowball’. Public invitations were sent through different social networks on digital platforms such as E-mails, WhatsApp, Facebook, and Instagram to recruit individuals able to participate in the research.

Data collection was carried out using an electronic form consisting of 21 questions, constructed using the Google Forms Online survey management tool.

This instrument was structured into three groups of questions: (1) socioeconomic, demographic, and anthropometric characteristics, related to monthly family income (in Brazilian currency—reais), age, sex, education level, neighborhood in which they lived, and family composition, body weight, and height reported by the interviewee; (2) food consumption indicators—related to eating practices regarding the use of food delivery apps and the consumption of specific food items and the habit of preparing meals at home.

As for exposure to the digital food environment, the use of digital apps for purchasing food was queried. Therefore, categories were developed to investigate the way in which food prepared outside the home was ordered, defined as follows: I never order; I order by phone call; websites or social networks (Facebook, Instagram, WhatsApp); or through delivery apps (for example, Ifood, Uber Eats, Rappi, or specific apps from a restaurant or a large chain).

The categories of websites, social networks, and applications included different digital platforms constituting the digital food environment and were presented as “online—delivery services for food prepared outside the home”. In this study, individuals were categorized into those who used delivery apps and those who did not use delivery apps to order food prepared outside the home. Therefore, individuals who used websites and social networks were allocated to the group that did not use apps.

Food items were classified into healthy and unhealthy eating markers. For this purpose, food consumption indicators from the Surveillance System of Risk and Protective Factors for Chronic Diseases by Telephone (VIGITEL) survey from the Ministry of Health were used, with the groups including beans, fresh fruits and vegetables, and/or legumes being considered as markers of healthy eating, whereas the groups that included hamburger and/or cold cuts; sugary drinks; instant noodles, packaged snacks, or savory crackers; and stuffed cookies and sweets were considered as markers of unhealthy eating.

Using weight and height measurements, nutritional status was classified according to the body mass index (BMI) cut-off points, according to the World Health Organization, WHO (1998), with a BMI below 24.9 kg/m^2^ being considered normal weight, a BMI between 25.0 and 29.9 kg/m^2^ considered overweight, and a BMI ≥ 30 kg/m^2^ indicating obesity.

Descriptive analyses of the population’s socioeconomic characteristics and food consumption practices were described in absolute and relative numbers according to application use. The differences between those who used and those who did not use the apps were tested using Pearson’s chi-square test. Logistic regression models were developed to evaluate the association between the frequency of the consumption of food markers of healthy and unhealthy eating and delivery applications for food prepared outside the home, with the use of the application as an exposure variable and the consumption of each food group as an outcome variable. The models were developed for each marker of healthy and unhealthy eating, with the frequency rarely being the reference category for food consumption.

The significance level adopted was 5%. The analyses were conducted with the SAS software, online version, https://www.sas.com/zh_cn/home.html. The study was submitted to the Research Ethics Committee (REC) and was conducted according to the principles established by the National Health Council (CNS) in Resolution Number 466/2012 and approved by the REC, under number 4,059,557.

## 3. Results

Most participants were female, and the sample mainly comprised individuals with a high education level and family income. Most respondents reported using home delivery services to purchase food prepared outside the home (91%). Of the interviewees who used the services, 78.1% used delivery apps to buy food prepared outside the home (Table 1).

The mean age of the individuals was 38.6 years, being lower in those who used applications (34.6 years) compared to those who did not use them (42.6 years) (*p*-value < 0.001).

The presence of adolescents at home was higher among individuals who did not use application services (28.8%) when compared to individuals who used them (23.9%) (*p*-value < 0.0328) (Table 1).

The habit of preparing food daily at home was more common among individuals who did not use apps to purchase food (89.8%) when compared to those who did (74.4%) (*p*-value < 0.0001).

Table 2 shows that the majority of individuals who rarely consumed food prepared outside the home did not use apps to purchase food (61.6%), compared to those who used these services (47.3%).

It was observed that the daily frequency of the consumption of beans, fresh fruits, and/or vegetables was higher for individuals who did not use delivery apps to purchase food. On the other hand, the weekly frequency of the consumption of hamburgers and/or cold cuts, sugar-sweetened beverages, instant noodles, packaged snacks or savory biscuits, sandwich cookies, and sweets was higher for individuals who used these online delivery services for food prepared outside the home (Table 2).

The odds ratio of the individual consuming beans, fresh fruits, and vegetables daily decreased due to the use of applications. The odds ratio of an individual consuming hamburgers and/or cold cuts and sugar-sweetened beverages daily and weekly increased for individuals who used delivery apps. This relationship remained independent of sex, age, and level of schooling (Table 3).

## 4. Discussion

In this study, we investigated whether the use of delivery apps for food prepared outside the home is related to food consumption practices and observed that individuals who were exposed to food offers in the digital food environment through delivery apps were less likely to choose the daily consumption of foods considered markers of healthy eating and, in parallel, had a greater chance of weekly and daily consumption of foods considered markers of unhealthy eating.

Our findings corroborate other studies on the consumption of food prepared outside the home offered by food delivery services, which show there is a high frequency of fast food orders, which are commonly characterized by items of low nutritional quality, among orders in online applications [6,7]. Our data contribute to studies on the phenomenon of increased exposure to DFE and its influence on purchasing practices for food prepared outside the home and consumer behavior.

In an exploratory study developed to assess the availability of food and the use of marketing strategies in two food delivery applications, carried out in Brazil, it was found that there is greater availability of ultra-processed beverages when compared to natural ones, such as water and fruit juices. Moreover, offers of ready-to-eat items such as pizzas, sandwiches, and fried snacks were more available when compared to meals based on natural or minimally processed foods, such as vegetables [8].

Furthermore, the authors highlighted a greater variety of items such as ice cream and sweets than fresh fruit, and the high appeal for the consumption of ultra-processed foods through photos and promotions [8]. In agreement, we observed in our sample a greater frequency of the consumption of items considered to be unhealthy based on the eating practices of delivery app users.

The digital food environment has changed the way people access food, with the increase in places that started to sell food further away from homes, considering that digital application platforms provide a greater number of store and restaurant options than previously observed in the neighborhood food environment [9,10].

Given this scenario, the digital food environment is related to an obesogenic environment, as it shows characteristics similar to a ‘food swamp’, in which there is a high presence of foods considered markers of unhealthy eating [5]. The characteristics of food marketed via food delivery apps are similar to food often consumed at outside home establishments. In Brazil, ultra-processed foods, such as fast foods and soft drinks, are widely consumed. As the frequency of consumption outside the home increased, the contribution of ultra-processed foods, especially sugary drinks and ready meals, increased, and the consumption of ultra-processed foods was higher when consumption took place outside the home [11].

One important issue of our study was that the collection period was during the social isolation measures of the COVID-19 pandemic, and the social isolation measures contributed to the increased exposure to digital platforms in the digital food environment. In this context, several food sales establishments started selling on digital platforms and using marketing strategies to attract customers during the pandemic [12].

However, the habit of purchasing food via online applications did not decrease after the end of the social isolation measures caused by the COVID-19 pandemic. Studies have shown that this behavior was established even with the return to activities and established itself as a new way of accessing food [5,12,13]. Considering the isolation period, our findings corroborate those of the CONVID Behavior Research study, carried out between 24 April and 24 May 2020, with a sample of 45,161 adult individuals living in Brazil, which showed a reduction in the frequency of the consumption of foods considered markers of healthy eating and, in parallel, an increase in the consumption of foods that are markers of unhealthy eating [13].

In the current global context, the use of digital technologies is a growing phenomenon in the daily practices of individuals. Therefore, it becomes relevant to know the dimensions of the digital food environment and how they interact with food acquisition behavior in the digital scope and the transformations in food consumption patterns. Our study stands out for providing an initial overview of this phenomenon in a Brazilian city [5,12,13].

The scenario of the expansion of digital configurations for the sale of food prepared outside the home suggests that the food industry adapted to the demands throughout the health crisis through online sales strategies during social isolation. Furthermore, the habit of purchasing food through delivery apps can become part of the individuals’ daily practice, not only because of the availability but also because of convenience, saving time and effort [12,13].

There are still few studies elucidating the strategies and influences of the digital food environment on people’s food consumption practices. A concern with the habit of using applications to purchase food is that digital platforms have been identified as an environment in which there is a high availability of foods considered unhealthy, such as ultra-processed items [8,9], which commonly have low nutritional quality and high energy density.

Overall, items such as fast-food snacks and culinary preparations based on processed and ultra-processed foods are considered markers of unhealthy eating, as they are options with high caloric density and low nutrient content, in general, with high amounts of sodium, saturated fats, trans fatty acids, and sugars, which are related to an increased risk of developing chronic non-communicable diseases (NCDs), such as obesity, cardiovascular diseases, and diabetes [7,14,15]. These diseases raise great concern for public health worldwide considering their combination of environmental and behavioral factors, in addition to the physiological and genetic aspects [16].

It is important to highlight that the sample in this study differs from the socioeconomic characteristics expected for the population of the state of Ceará, considering that the individuals had a high level of education and high family income. However, this socioeconomic profile was also verified in a study carried out using an online form, with 19,378 adult individuals, on the prevalence and frequency of the use of online delivery services for food prepared outside the home with data from five countries: Australia, Canada, Mexico, United Kingdom, and the USA. The researchers observed that respondents with a higher level of education were more likely to use apps to purchase food, also finding that men tended to use more apps to order food and cook less than women [17]. 

From another perspective, at the interface of food systems and the impact of climate and environmental changes, the food production chain is closely linked to these global changes, which affects the availability and cost of food production. Moreover, there is a growing intention to reduce the carbon footprint of food production, aiming at diet quality and food safety, considering the need for food production that also guarantees safety and sustainability [18].

A positive aspect of our study is that it demonstrated that individuals who did not use delivery apps showed greater adherence to the habit of cooking at home, in addition to a greater frequency of daily consumption of beans, fresh fruits, and vegetables, considered markers of healthy eating.

The habit of cooking at home is a focus of health promotion efforts, considering that it is associated with improved diet quality. In this context, there is an opportunity to encourage culinary skills that adopt food choices that are not only healthy for human health but also more sustainable for the health of the planet, such as for instance, the use of alternatives to red meat and a diet based on plant sources [19].

## 5. Conclusions

We found that the use of delivery applications is related to users’ eating practices through the acquisition of food in the digital scope. We also observed a relationship between exposure to the DFE from delivery apps with the weekly frequency of the consumption of items considered to be markers of unhealthy eating. Conversely, we found that the odds ratio of an individual consuming items considered markers of healthy eating on a daily basis decreased with the use of apps for the delivery of food prepared outside the home. The present study highlights the phenomenon of the rise in the use of delivery applications to purchase food prepared outside the home and points to the relevance of studies that elucidate the influences of the digital food environment on the transition of people’s eating patterns. These results highlight the need for actions aimed at regulating food offers at food delivery apps and the need for nutritional educational strategies in the digital food environment. Also, it is necessary to stimulate more healthy food options by food establishments. 

## Figures and Tables

**Table 1:** Characteristics of the population according to the use of delivery applications for food prepared outside the home. Fortaleza, Ceará, Brazil. 2020.

Variables	Did Not Use Delivery Applications		Used Delivery Applications		*p*-Value
	N	%	N	%	
Sex Male Female					<0.1338
	108	23.4	333	20.2	
	354	76.6	1.318	79.8	
Education level Complete Elementary school or incomplete High School Complete High School or complete Higher Education Complete Graduate School					<0.0154
	7 197 258	1.5 42.6 55.8	11 810 830	0.7 49.0 50.3	
Family Income <3500 3.500 to 7.000 7.000 to 12.540 >12.540					<0.8439
	114	24.7	424	25.7	
	107	23.2	402	24.3	
	124 117	26.8 25.3	414 411	25.1 24.8	
Presence of children					0.3432
Yes	110	23.8	429	26.0	
No	352	76.2	1222	74.0	
Presence of adolescents					0.0328
Yes	133	28.8	395	23.9	
No	329	71.2	1256	76.1	
Nutritional status					<0.9725
Normal weight	232	50.2	834	50.5	
Overweight	156	33.7	560	33.9	
Obesity	74	16.0	257	15.5	

**Table 2:** Food consumption practices according to the use of delivery applications for food prepared outside the home. Fortaleza, Ceará, Brazil. 2020.

Variables	Did Not Use Delivery Applications		Used Delivery Applications		*p*-Value
	N	%	N	%	
Frequency of consumption of food prepared outside the home					<0.0001
Rarely	300	64.9	401	24.3	
Weekly	143	31.0	1.156	70.0	
Daily	19	4.1	94	5.7	
Frequency of preparing food at home					<0.0001
Rarely	7	1.5	34	2.1	
Weekly	40	8.7	389	23.5	
Daily	415	89.8	1228	74.4	
Beans					<0.0001
Rarely	63	13.6	297	18.0	
Weekly	210	45.5	941	57.0	
Daily	189	40.9	413	25.0	
Fresh fruits					<0.0001
Rarely	31	6.7	171	10.4	
Weekly	155	33.6	841	50.9	
Daily	276	59.7	639	38.7	
Vegetables and/or legumes					<0.0001
Rarely	46	10.0	251	15.2	
Weekly	211	45.7	917	55.5	
Daily	205	44.3	483	29.3	
Hamburger and/or cold cuts					<0.0001
Rarely	271	58.7	669	40.5	
Weekly	176	38.1	886	53.7	
Daily	15	3.3	96	5.8	
Sugar-sweetened beverages					<0.0001
Rarely	256	55.4	629	38.1	
Weekly	148	32.0	744	45.1	
Daily	58	12.6	278	16.8	
Instant noodles, packaged snacks, and savory biscuits					0.0036
Rarely	332	71.9	1048	63.5	
Weekly	122	26.4	569	34.5	
Daily	8	1.7	34	2.0	
Sandwich cookies and sweets					<0.0001
Rarely	239	51.7	599	36.2	
Weekly	189	40.9	881	53.4	
Daily	34	7.4	171	10.4	

**Table 3:** Odds ratio (OR) and 95%CI for the association between the frequency of consumption of food markers of healthy and unhealthy eating and the use of delivery apps for food prepared outside the home. Fortaleza, Ceará, Brazil. 2020.

Variable	Non-Adjusted OR (95%CI)	OR Adjusted for Age and Sex (95%CI)	OR Adjusted for Age, Sex and Level of Schooling (95%CI)
Beans			
Rarely	1.00	1.00	1.00
Weekly	0.95 (0.70–1.30)	1.04 (0.74–1.46)	1.05 (0.75–1.48)
Daily	0.46 (0.34–0.64)	0.43 (0.306–0.62)	0.44 (0.31–0.63)
Fresh fruits			
Rarely	1.00	1.00	1.00
Weekly	0.98 (0.65–1.50)	1.09 (0.70–1.70)	1.08 (0.69–1.68)
Daily	0.42 (0.28–0.63)	0.61 (0.40–0.95)	0.60 (0.38–0.92)
Vegetables and/or legumes			
Rarely	1.00	1.00	1.00
Weekly	0.80 (0.56–1.13)	0.89 (0.61–1.30)	0.87 (0.60–1.28)
Daily	0.43 (0.30–0.62)	0.51 (0.35–0.76)	0.50 (0.34–0.74)
Hamburger and/or cold cuts			
Rarely	1.00	1.00	1.00
Weekly	2.04 (1.65–2.53)	1.72 (1.36–2.18)	1.73 (1.36–2.19)
Daily	2.59 (1.48–4.55)	2.14 (1.16–3.96)	2.13 (1.15–3.94)
Sugar-sweetened beverages			
Rarely	1.00	1.00	1.00
Weekly	2.05 (1.63–2.57)	1.70 (1.33–2.19)	1.76 (1.37–2.27)
Daily	1.95 (1.42–2.68)	1.52 (1.08–2.14)	1.61 (1.14–2.27)
Instant noodles, packaged snacks, and savory biscuits			
Rarely	1.00	1.00	1.00
Weekly	1.48 (1.17–1.86)	1.13 (0.88–1.45)	1.14 (0.89–1.47)
Daily	1.35 (0.62–2.94)	0.83 (0.37–1.84)	0.84 (0.38–1.87)
Sandwich cookies and sweets			
Rarely	1.00	1.00	1.00
Weekly	1.86 (1.50–2.31)	1.44 (1.14–1.83)	1.42 (1.12–1.80)
Daily	2.01 (1.35–3.00)	1.32 (0.872.03)	1.30 (0.85–1.99)

## Data Availability

The datasets presented in this article are not readily available due to ethical restrictions. Requests to access the datasets should be directed to the corresponding author.
